# Ibogalogs reduce acute and chronic headache-like behaviours in rodents by different co-modulatory mechanisms involving 5-HT_1_ and 5-HT_2_ receptor subtypes

**DOI:** 10.21203/rs.3.rs-10924055/v1

**Published:** 2026-09-18

**Authors:** Shuo-Fu Chen, Hugo R. Arias, Alejandro Abraham, Daniel Wacker, Tally M. Largent-Milnes

**Affiliations:** a Graduate Interdisciplinary Program in Neuroscience, University of Arizona, Tucson, AZ, USA; b Comprehensive Center for Pain & Addiction (CCPA), University of Arizona Health Sciences, Tucson, AZ, USA; c Department of Pharmacology and Physiology, Oklahoma State University College of Osteopathic Medicine, Tahlequah, OK, USA.; d Department of Pharmacological Sciences and Department of Neuroscience, Icahn School of Medicine at Mount Sinai, New York, NY, USA; e Department of Pharmacology, University of Arizona, Tucson, AZ, USA

**Keywords:** Ibogalogs, migraine, 5-HT_1_ receptors, 5-HT_2_ receptors

## Abstract

The main objective of this work is to assess whether ibogalogs decrease headache-like behaviors in rats using the cortical spreading depression (CSD) and medication overuse headache (MOH) models. The CSD and MOH results showed that tabernanthalog (TBG), ibogainalog (IBG), and ibogaminalog (DM506) significantly decreased allodynia induced by acute injection of KCl or repetitive administration of sumatriptan. The effect of ibogalogs in the CSD model was sex-independent. The observed effects lasted 6-8 h, with TBG showing comparatively higher efficacy in both models. We also assessed the potential role of serotonin type 2 receptor (5-HT_2_R) and 5-HT_1B/1D_R subtypes the effects of ibogalogs using selective antagonists such as ketanserin and GR127935, respectively. The antagonist effects were headache type-dependent: in acute headache, GR127935 inhibited ibogalogs’ activity during the first hour but subsequently enhanced their effects, whereas ketanserin enhanced their activities; in chronic headache, GR127935 abolished ibogalog’s effects during the first 3 h, whereas ketanserin inhibited IBG’s effect during the first 2 h but enhanced TBG’s effect. These results suggested that the antiheadache effects of ibogalogs are mediated by co-modulation of 5-HT_1_R and 5-HT_2_R subtypes. The functional results showed the ibogalogs activate 5-HT_1_R subtypes with the following selectivity: 5-HT_1F_R ~ 5-HT_1D_R > 5-HT_1B_R >> 5-HT_1E_R. Thus, we compared the activity of ibogalogs between wild type (WT) and 5-HT_1F_R knockout (KO) mice in the MOH model. The results in WT mice showed that ibogalogs increase the facial withdrawal threshold with the following efficacy: TBG > IBG > DM506. Moreover, ibogalog antiallodynic effects were reduced in KO mice after 4 h, supporting a role for the 5-HT_1F_R in the actions of ibogalogs in headache attenuation. In conclusion, ibogalogs significantly reduce acute and chronic headache-like behaviors by a co-modulatory mechanism between 5-HT_1_R and 5-HT_2_R subtypes.

## Introduction

Migraine is a complex, multi-phasic neurological disorder where one of the most important symptoms, throbbing head pain, is more severe and longer-lasting (up to several days) than common headaches[1]. Migraines can be triggered by different stimuli (e.g., stress, hormonal and environmental factors, and certain foods), and are typically accompanied by other symptoms, including nausea/vomiting, irritability, insomnia, and sensory hypersensitivity, interfering with daily activities. There are several medications for the treatment (e.g., triptans) and prevention (e.g., erenumab) of migraine attacks. One of the most effective treatments is based on the activation of different serotonin type 1 receptor subtypes (5-HT_1_R) (reviewed in [2, 3]). For instance, triptans activate the presynaptic 5-HT_1D_R, which inhibits dural vasodilation and neurogenic inflammation, as well as activate brainstem 5-HT_1B/1D_R subtypes, inhibiting trigeminal nuclei cell excitability, and vascular 5-HT_1B_Rs, inducing meningeal, dural cerebral, and pial vasoconstriction. Nevertheless, chronic use of triptans and other antimigraine drugs may induce secondary chronic headache, so called “rebound headache” [4-7]. These mechanisms supported the hypothesis that overstimulation of the trigeminovascular system is the culprit of migraine [8]. However, lasmiditan, a selective 5-HT_1F_R agonist, reduces migraine symptoms without vasoconstriction, supporting trigeminal inhibition as a critical mechanism [9], although it is no longer used clinically because of safety concerns about its side effects [10]. Notably, previous studies discovered 5-HT_1F_R agonist activity mediated by mianserin and mirtazapine, two antidepressants with efficacy in the prophylaxis and treatment of migraine, further highlighting the underappreciated role of this receptor in antimigraine activity [11-13].

**Figure 1.**
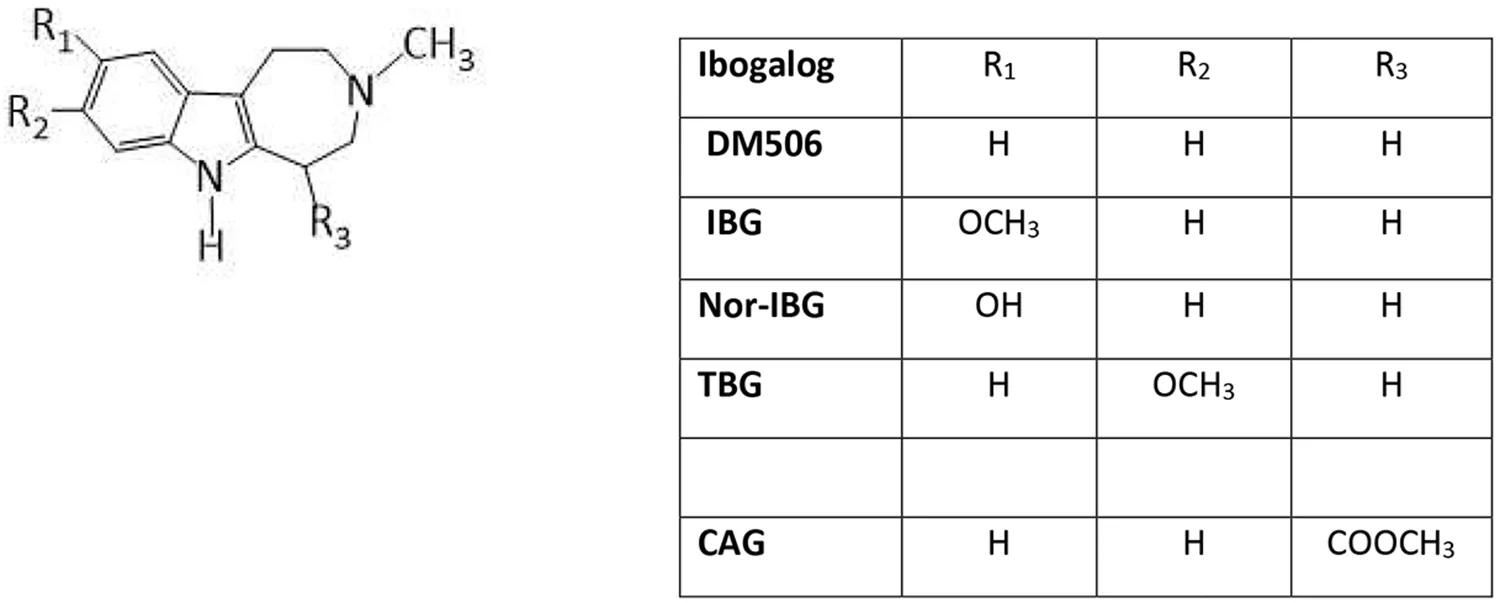
Molecular structures for DM506 (ibogaminalog), IBG (ibogainalog), and TBG (tabernanthalog).

Ibogalogs are synthetic compounds that maintain part of the structure of iboga alkaloids (see molecular structures in Fig. 1) but present different pharmacological profiles at metabotropic serotonin (5-HT) receptors. These compounds modulate the activity of different metabotropic serotonin receptors, including the 5-HT_1_R and 5-HT_2_R subfamilies [14-21]. Pre-clinical studies in rodents showed that ibogalogs induce antidepressant- and anxiolytic-like activity [16, 18] as well as reduce chronic pain [15, 17, 21], which might be important for a potential antimigraine activity. Our laboratory recently determined that ibogalogs also induce antiseizure activity in rodents [20]. The main mechanism of action of these behavioral effects seems to be centered in the activation of 5-HT_2A/2C_Rs. Seizures, similar to migraine, induce cortical spreading depression (CSD), a wave of cortical hyperactivity followed by a wave of inhibitory activity (reviewed in [22]). Although we do not know the exact mechanism supporting seizure and migraine comorbidity, it is possible that ibogalogs can also attenuate migraine symptoms *via* 5-HTR modulation. In this regard, studies herein assessed whether TBG, IBG, and ibogaminalog (DM506) reduce migraine-like periorbital allodynia using rat models of CSD and medication overuse headache (MOH) induced by sumatriptan [4-7]. To assess the potential role of different 5-HT receptor subtypes, selective antagonists, including ketanserin (5-HT_2_R) and GR127935 (5-HT_1B/1D_R) [23, 24] as well as 5-HT_1F_R knockout (KO) mice were employed in behavioral experiments. The functional activities of several TBG, IBG, and DM506 at the 5-HT_1B_R, 5-HT_1E_R, and 5-HT_1F_R subtype were also assessed.

## 2. Material and Methods

### 2.1. Materials

Recombinant HEK293 cells overexpressing each human 5-HT_1B_R, 5-HT_1E_R, and 5-HT_1F_R, HTRF IP-One G_q_ Detection Kit, and Unifilter^®^ GF/C plates were obtained from Revvity (Waltham, MA, USA; Zurich, Switzerland). The HEK293-T cell line was obtained from American Type Culture Collection (ATCC^®^) (Manassas, VA, USA). NIH/3T3 cells, serotonin hydrochloride (5-HT), pargyline hydrochloride, and ascorbic acid were purchased from Merck (Buchs, Switzerland). Sumatriptan was obtained from Sigma-Aldrich (St. Louis, MO, USA) and isoflurane was purchased from VetOne (IL, USA). Ketanserin tartrate was purchased from Adooq Bioscience LLC (Irvine, CA, USA), and GR127935 hydrochloride from Bio-techne Tocris (Bristol, UK). The GloSensor was purchased from Promega (Madison, WI, USA). Polyethyleneimine was purchased from Merck (Buchs, Switzerland) and Alfa Aesar (Ward Hill, MA, USA). Poly-l-lysine-coated clear-bottom 384-well plates were obtained from Greiner Bio One (Monroe, NC, USA). D-luciferin was obtained from Gold Biotechnology Inc. (St. Louis., MO, USA). Opti-MEM plates were purchased from Thermo Fisher Scientific (Waltham, MA, USA). Scintillation vials (6 mL), Ultima Gold and MicroScint-20 or -PS scintillation cocktails were obtained from PerkinElmer (Schwerzenbach, Switzerland). Penicillin, streptomycin, Dulbecco’s modified Eagle medium (DMEM), and fetal bovine serum (FBS) were purchased from Corning Cellgro (Manassas, VA, USA). Ibogainalog hydrochloride (IBG), nor-IBG (base), ibogaminalog hydrochloride (DM506), tabernanthalog fumarate (TBG), and catharanthalog (base) (CAG) were synthesized by Ambeed, Inc. (Arlington Hts, IL, USA), according to previously described protocols [14, 25]. Salts, bases, buffers, and solvents were purchased from commercial suppliers and were used as received.

### 2.2. Animals

Adult female and male Sprague Dawley rats (7–8 weeks on arrival, 150–250 g) were purchased from Envigo (Indianapolis, IN) and wild-type C57Bl/6J (WT) mice (7–8 weeks on arrival) were obtained from The Jackson Laboratories (Bar Harbor, ME). Cohorts were habituated to the vivarium for at least 1 week and were handled daily to mitigate stress. Male and female 5-HT_1F_R KO mice (9-12 months) were kindly provided by Dr. Rick Schnellmann. Animals were housed in groups in standard cages with free access to standard laboratory diet and tap water. All animals were kept in a ventilated room, at a temperature of 22 ± 1 °C, under a 12-h light/12-h dark cycle (light between 7:00 a.m. and 7:00 p.m.). All experimental procedures were conducted during the 12-hour light cycle, under the National Institutes of Health Guide for the Care and Use of Laboratory Animals, the policies and recommendations of the International Association for the Study of Pain, and with approval from the Regional Ethics Committee for Animal Experimentation and the Institutional Animal Care and Use Committee of the University of Arizona (#17-223).

### 2.3. Effect of ibogalogs on cortical spreading depression (CSD)-induced periorbital allodynia in rats

To induce periorbital allodynia, a symptom commonly found in patients suffering from migraine [26, 27], a protocol adapted from previously established methods [7, 28, 29] was used. Briefly, male and female rats were anesthetized *via* intraperitoneal injection of a ketamine/xylazine/acepromazine cocktail (45/5/2 mg/kg). Vital signs, including heart and respiratory rates, skin color, and responsiveness to toe pinch, were continuously monitored to ensure adequate anesthesia depth and physiological stability throughout the surgical procedure. Animals were secured in a stereotaxic apparatus (Stoelting Co.), and a midline incision (approximately 1.5–2 cm) was made to expose the skull. Connective tissue was gently cleared as needed. A small burr hole was drilled at coordinates −6.0 mm posterior and −3.0 mm lateral to bregma (Ram Productions MICROLAB 350/450), taking care not to penetrate the dura. A 22-gauge stainless steel guide cannula (#C313G, Plastics One) was positioned just above the dura (0.5 mm from the skull surface) and affixed with cyanoacrylate adhesive. Two additional anchoring holes were drilled rostral and contralateral to the cannula for stainless-steel screws (#MPX-080–3F-1 M; Small Parts). The assembly was secured with dental acrylic, and a dummy cap (#C313DC; Plastics One) was inserted to maintain cannula patency.

For cortical stimulation, lightly restrained awake rats received a focal cortical injection through the implanted guide cannula using a Hamilton microsyringe (30 GA, #80308 701 SN; Hamilton Company). The injector extended 1.0 mm beyond the guide cannula to reach the occipital cortex and delivered a 0.5 μL bolus of 1 M KCl solution, prefiltered through a 0.2 μm syringe filter. The KCl injection was designated as t = 0 min for all subsequent behavioral assessments.

Rats were grouped based on their postsurgical baseline to ensure equivalent pre-injection thresholds (6–8 g) at 7 to 10 days. Animals with post-cannulation, pre-cortical injection thresholds of 6–8 g were included, whereas animals with a periorbital withdrawal threshold at baseline or post-surgical baselines of < 6 g were excluded according to protocol. Rats were acclimated to the testing box 1 h prior to evaluation of periorbital mechanical allodynia with calibrated von Frey filaments (1, 1.4, 2, 4, 6, and 8 g) using the up-down method [30, 31].

Rats were then randomly assigned to four groups and administered (i.p.) one of the following: 10 mg/kg IBG hydrochloride [equivalent to 8.6 mg/kg IBG (base)], 25 mg/kg DM506 hydrochloride [equivalent to 21 mg/kg DM506 (base)], 40 mg/kg TBG fumarate [equivalent to 26.6 mg/kg TBG (base)] (all dissolved in 1% DMSO, 1% Tween 80, and 0.9% saline; [18]), or vehicle. To assess the role of specific receptor subtypes, rats were injected (i.p.) 1 mg/kg ketanserin (5-HT_2_R antagonist; [23]) or 0.1 mg/kg GR127935 (5-HT_1B/1D_R antagonist; dose modified from [32]) 30 min prior to ibogalog treatment.

### 2.4. Effect of ibogalogs on overuse headache (MOH) in female rats

MOH was induced in female rats by sustained sumatriptan infusion [28]. Only animals with a periorbital withdrawal threshold of 6 g or greater before surgery were included. Sustained infusion was attained using osmotic minipumps (model 2001) (Alzet, Cupertino, CA, USA) with a flow rate of 1 μL/h for 7 d. The minipumps were implanted subcutaneously under anaesthesia with isoflurane (flow rate = 2 L/min in 100% O_2_). A 4–5 mm incision was made between the shoulder blades, and the osmotic minipump was inserted under the skin. The incision site was closed with wound clips. The day of the pump implant was considered as day 0. Sumatriptan was administered at 0.6 mg/kg/day.

On day 7, rats were acclimated to the testing box for 1 h prior to the evaluation of periorbital mechanical allodynia using calibrated von Frey filaments (1, 1.4, 2, 4, 6, and 8 g), followed by post infusion baseline assessment of periorbital allodynia.

Ibogalog and antagonist treatments were the same as that described in section 2.3. Following drug administration, the facial withdrawal threshold was measured at baseline and at 20, 40, 60, 90, 120, 180, 240, 300, 360 min, and 24 h post-injection, using a modified version of the Dixon up-down method [29]. von Frey filaments were applied perpendicularly to the midline of the forehead at the level of the eyes with enough force to cause the filament to slightly bend while held for 5 s. A response was indicated by a sharp withdrawal of the head, vocalization, or severe batting at the filament with attempts to bite it.

### 2.5. Effect of ibogalogs on overuse headache (MOH) in wild type and 5-HT_1F_R knockout mice

Given that ibogalogs activate the 5-HT_1F_R with high potency (Table 1), we evaluated their efficacy against chronic headache using the MOH model in WT and 5-HT_1F_R KO mice (n = 7 per strain, split between male and female in each treatment). After 5 days of habituation and exposure to the environment and monofilaments, periorbital mechanical thresholds were evaluated using a series of von Frey filaments (0.008, 0.04, 0.07, 0.16, 0.4, 0.6, 1, and 2 g). Only animals with a periorbital withdrawal threshold of ≥1.4 g were included in the study. MOH was induced in both female and male mice through daily sumatriptan injection (0.6 mg/kg, i.p.) for seven consecutive days. Periorbital mechanical thresholds were evaluated daily from day 7 to 14. In addition, mice received ibogalog injections on days 7, 9, 11, and 13, according to a within-subjects, randomized Latin square design following the completion of daily threshold assessments [33, 34].

### 2.6. Functional activity of ibogalogs at 5-HT_1B/1E/1F_R subtypes

The pharmacological activity of ibogalogs was assessed at the 5-HT_1B_R, 5-HT_1E_R, and 5-HT_1F_R using previously published TRUPATH reporter constructs [35]. Assays were essentially performed as previously described [13, 36]. In brief, each receptor, as well as RLuc8-Gαi1, Gβ3, and eGFP-Gγ9 constructs were transfected with polyethyleneimine into HEK293-T cells using a ratio of 1:1:1:1 (human 5-HT_1_:Gα:Gβ:Gγ). Typically, 6-7 million cells in DMEM supplemented with 1% (v/v) dFBS in a 10 cm plate were transfected with a total of 8 μg of DNA. The next day, 20,000 cells/well were plated into poly-L-lysine-coated white-bottom 384-well plate in DMEM supplemented with 1% dFBS. The following day, the media was aspirated and the cells were washed with HBSS supplemented with 20 mM HEPES (pH 7.4). 45 μL of drug solutions at indicated concentrations in HBSS supplemented with 20 mM HEPES (pH 7.4), 0.1% (w/v) FBS, and 0.01% (w/v) ascorbic acid were then added to the cells. Cells were incubated for 30 min at 37 °C followed by addition of 15 μL of freshly prepared 30 μM coelenterazine 400a. Plates were then immediately read in a Victor NIVO plate reader (Perkin Elmer) with 395 nm (RLuc8-coelenterazine 400a) and 510 nm (GFP2) emission filters at integration times of 1 s per well. Bioluminescence Resonance Energy Transfer (BRET) ratios were computed as the ratio of the GFP2 emission to RLuc8 emission.

### 2.7. Statistical Analysis

The experimental data were analyzed using the Prism software (GraphPad 10.4.0, Software Inc., La Jolla, CA, USA) and R (version 4.4.2). Time-course behavioral data in rats were analyzed using mixed-design two-way analysis of variance (ANOVA) with drug treatment and time as factors. For transgenic mice, data were analyzed using repeated-measures (RM) two-way ANOVA to account for the within-subjects design. These analyses were performed separately for each antagonist condition. The area under the curve (AUC) was calculated from the time-course data. For rats, AUC values were analyzed separately under each antagonist condition using one-way ANOVA to compare the efficacy of different treatments. To further assess sex differences, AUC data from all antagonist conditions were combined and analyzed using a three-way ANOVA with drug, antagonist condition, and sex as between-subject factors. Similarly, drug effects in mice were compared within each strain using RM one-way ANOVA. To combine the genotype factor, AUC data were processed using a two-way ANOVA. To integrate all behavioral data and analyze the most efficient time points for the interaction between drug and antagonist condition (in rats) or genotype (in mice), a mixed three-way ANOVA with factors of ibogalogs, time, and antagonist condition or genotype was performed. Tukey’s post-hoc test was applied following significant main or interaction effects. To quantify the proportion of variance contributed by each factor and its interactions, partial eta squared ($\eta_{p}^{2}$) was calculated as an indicator of effect size. Unpaired t-tests were used to compare the behavioral differences between the antagonist-alone group and the vehicle control in rats, as well as the differences between WT and KO mice receiving vehicles. Additionally, for the functional assays, unpaired t-test was used to compare the slopes of each ibogalogs. All data points are displayed as the mean ± SEM, and statistical significance was set at p < 0.05; when appropriate F-values are reported. Based on a priori power analysis (G*Power 3.1), the sample size was determined to provide 80% power to detect a 20% difference (α = 0.05) at a significance level in behavioral experiments.

## 3. Results

### 3.1.a. Ibogalogs reduce CSD-associated periorbital allodynia in rats

**Figure 2.**
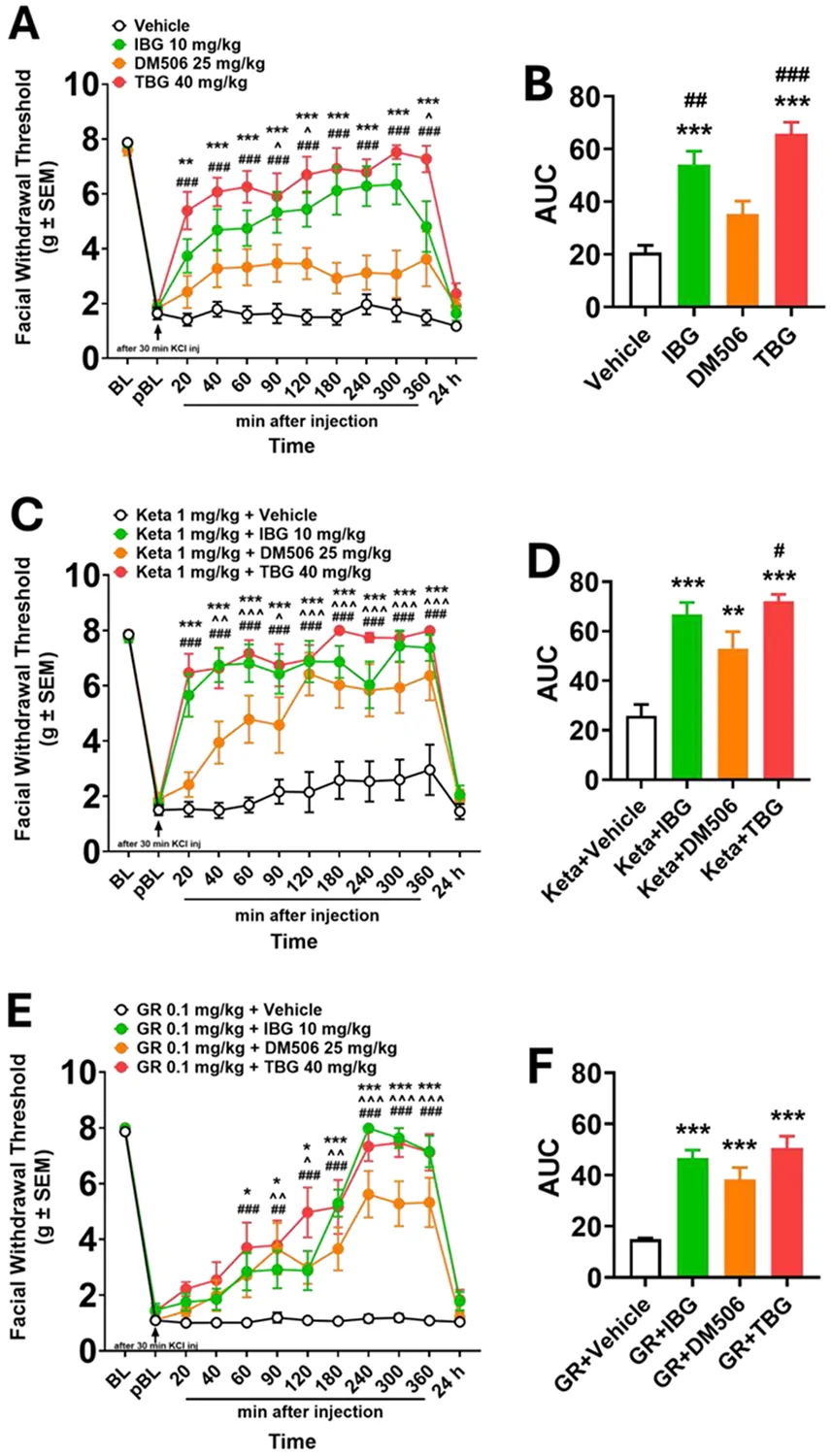
Effect of ibogalogs on periorbital allodynia induced by cortical KCl injection in rats. Pretreated rats were administered (i.p.) 8.6 mg/kg IBG (base) (•), 21 mg/kg DM506 (base) (•), 26.6 mg/kg TBG (base) (•), or vehicle (○). Facial withdrawal thresholds were measured at baseline (BL), post-cortical KCl injection baseline (pBL), 20, 40, 60, 90, 120, 180, 240, 300, 360 min, and 24 h post-injection. Data were analyzed by two-way ANOVA followed by Tukey’s post hoc test (mean ± SEM; n = 10 per group) for (A) effects of ibogalogs administration, (C) interaction between ketanserin (Keta) and ibogalogs, and (E) interaction between GR127935 (GR) and ibogalogs. Area under the curve (AUC) analyses performed by one-way ANOVA followed by Tukey’s post hoc test for (B) ibogalogs administration, (D) Keta and ibogalogs interaction, and (F) GR and ibogalogs interaction within 24 h. Panels A, C, and E: IBG *vs* vehicle, *p <0.05, **p <0.01, ***p <0.001; DM506 *vs* vehicle, ^p <0.05, ^^p <0.01, ^^^p <0.001; TBG *vs* vehicle, ^#^p <0.05, ^##^p <0.01, ^###^p <0.001. Panels B, D, and F: ibogalogs *vs* vehicle, **p <0.01, ***p <0.001; TBG and IBG vs DM506, ^#^p <0.05, ^##^p <0.01, ^###^p <0.001.

Cortical KCl–induced periorbital allodynia was assessed in male and female rats to model CSD (Fig. 2). In both sexes, cortical injection of KCl (0.5 μL, 1 M) produced periorbital allodynia within 30 min, and facial allodynia persisted for 24 h compared to rats receiving vehicle injections (Fig. 2A). To assess whether ibogalogs reduce KCl-induced facial allodynia, rats were administered TBG [26.6 mg/kg (base)], IBG [8.6 mg/kg (base)], DM506 [21 mg/kg (base)], or vehicle. Two-way ANOVA analysis of the results revealed significant main effects of drug [F_(3,36)_ = 20.416; p < 0.001] and time [F_(11,396)_ = 54.367; p <0.001], as well as a significant drug-by-time interaction [F_(33,396)_ = 5.828; p <0.001]. Post-hoc Tukey’s tests indicated that, compared to the vehicle group, IBG and TBG robustly reduced CSD-induced periorbital allodynia over the 6-h observation period, whereas DM506 produced only a modest reduction. The analgesic effects of all compounds subsided at 24 h post-administration, indicating short-lasting effects. AUC analysis further confirmed group differences compared to the vehicle group [F_(3,36)_ = 20.811; p <0.001], exhibiting significantly higher AUC values than that for DM506 (Fig. 2B). Given that the dose for IBG was 3-fold lower than that for TBG, we can state that IBG is the most potent ibogalog.

### 3.1.b. Ketanserin enhances the anti-allodynic effects of ibogalogs in the CSD model

To assess the involvement of different 5-HT_2_R subtypes in the anti-allodynic actions of ibogalogs in the CSD model, 1 mg/kg ketanserin, a selective 5-HT_2A/2C_R antagonist [23], was administered (i.p.) 30 min prior to ibogalog treatment (Fig. 2C). Two-way ANOVA analysis of the results revealed significant main effects of drug [F_(3,36)_ = 16.628; p <0.001] and time [F_(11,396)_ = 66.324; p <0.001], as well as a significant drug-by-time interaction [F_(33,396)_ = 6.451; p <0.001]. Post-hoc Tukey’s tests showed that ketanserin potentiated the anti-allodynic responses produced by all three ibogalogs. More specifically, ketanserin significantly increased DM506 activity between 40 min (p <0.01) and 6 h (p <0.001) post-injection. AUC analysis further identified significant group differences [F_(3,36)_ = 17.302; p <0.001], and post-hoc comparisons indicated that ketanserin co-administration significantly increased the AUC for TBG (p <0.001), IBG (p <0.001), or DM506 (p <0.01), relative to the ketanserin (alone) group (Fig. 2D). Notably, ketanserin combined with TBG produced a greater AUC value than that combined with DM506 (p <0.05). However, ketanserin did not induce any effect *per se* compared to the vehicle group [AUC unpaired T-test: t_(18)_ = 0.956; p >0.05]. These findings suggest that the anti-allodynic activity of ibogalogs is, in part, modulated *via* 5-HT_2_Rs.

### 3.1.c. GR127935 reveals a biphasic effect of ibogalogs in reducing CSD-induced periorbital allodynia

To examine the role of 5-HT_1B/1D_R subtypes in ibogalog-induced analgesia in the CSD model, 0.1 mg/kg GR127935, a selective 5-HT_1B/1D_R antagonist [32], was administered (i.p.) 30 min prior to ibogalog treatment (Fig. 2E). Two-way ANOVA analysis of the results showed significant main effects of drug [F_(3,36)_ = 19.219; p <0.001] and time [F_(11,396)_ = 94.608; p <0.001], as well as a significant drug-by-time interaction [F_(33,396)_ = 7.611; p <0.001]. Post-hoc Tukey’s analyses indicated that GR127935 attenuated the anti-allodynic effects of ibogalogs during the first hour but subsequently enhanced their effects from 1 to 6 h post-treatment (p <0.001), which subsided at 24 h. AUC analysis further demonstrated significant group differences [F_(3,36)_ = 19.128; p <0.001], with GR127935 co-administration producing significantly higher AUC values for all ibogalogs compared to the GR127935 group (p <0.001; Fig. 2F). However, GR127935 did not induce any effect *per se* compared to the vehicle group [AUC unpaired T-test: t_(18)_ = 2.030; p >0.05]. These findings suggest that the anti-allodynic activity of ibogalogs is partially mediated by 5-HT_1B/1D_Rs.

### 3.1.d. Assessment of sex as a biological variable in drug- and antagonist-mediated modulation of CSD

**Figure 3.**
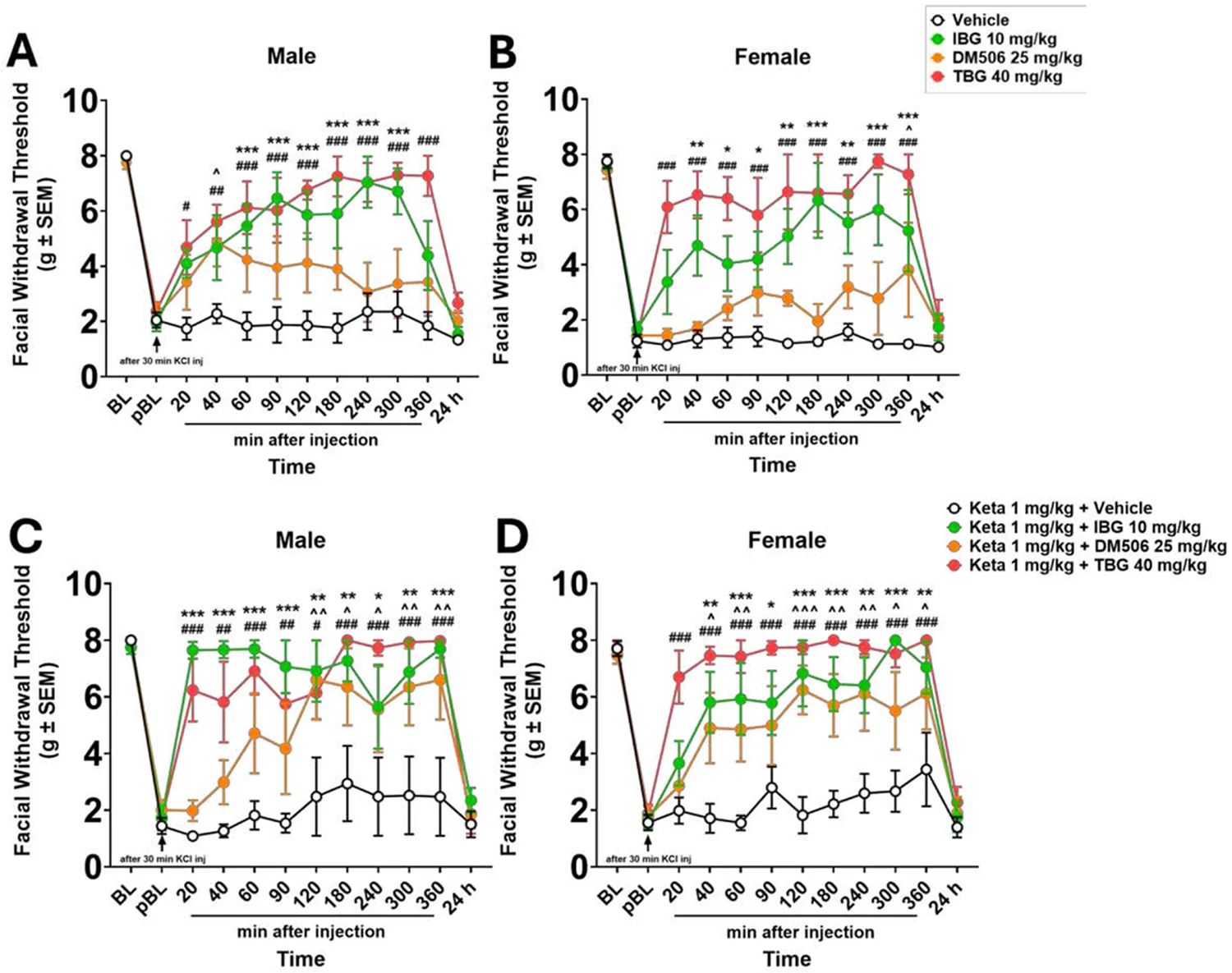

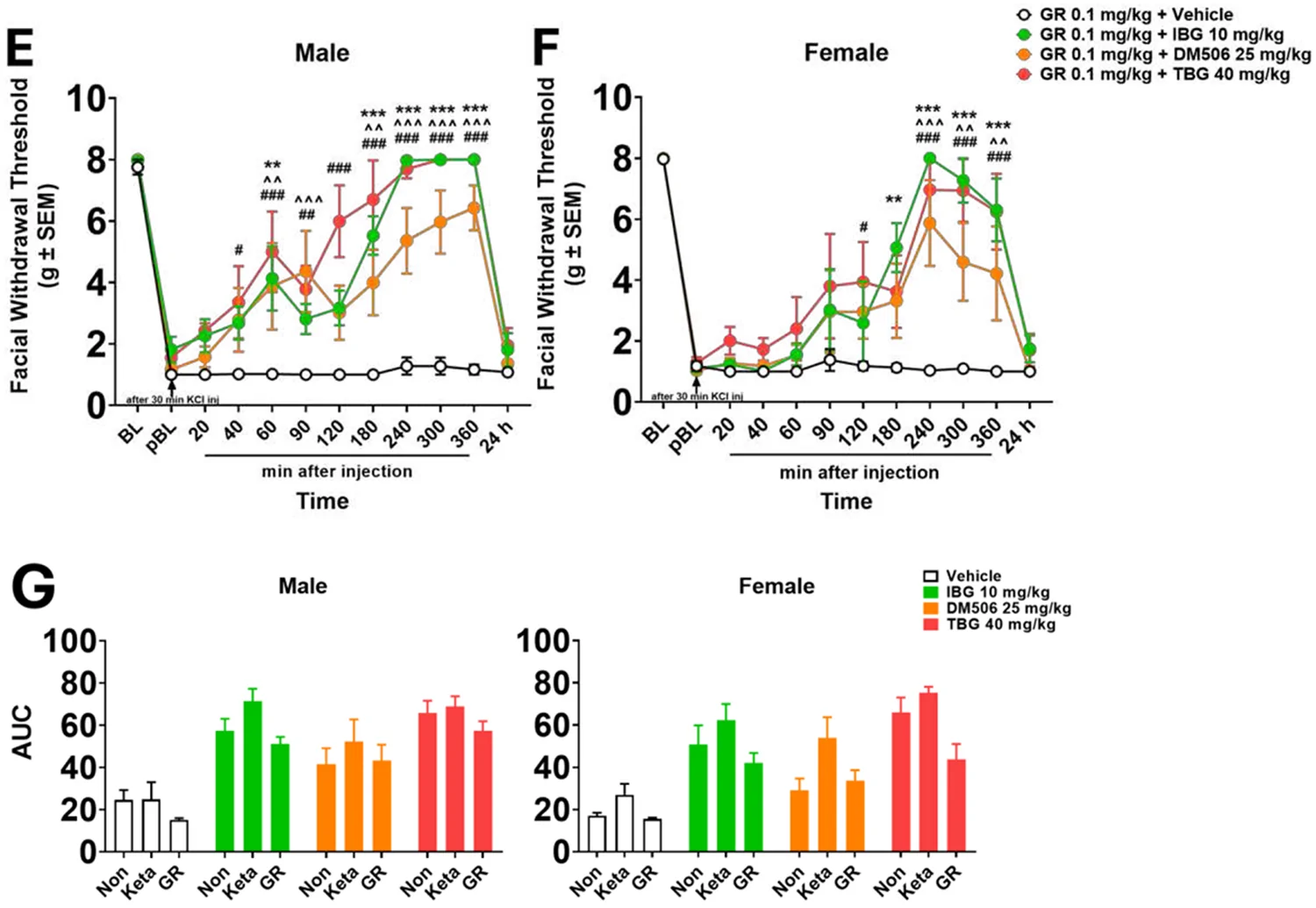
Effect of ibogalogs on periorbital allodynia induced by cortical KCl injection in male (A, C, and E) or female rats (B, D, and F). Pretreated rats were administered (i.p.) 8.6 mg/kg IBG (base) (•), 21 mg/kg DM506 (base) (•), 26.6 mg/kg TBG (base) (•), or vehicle (○). Facial withdrawal thresholds were measured at baseline (BL), post-cortical KCl injection baseline (pBL), 20, 40, 60, 90, 120, 180, 240, 300, and 360 min, and at 24 h post-injection. Two-way ANOVA followed by Tukey’s post hoc test (mean ± SEM; n = 5 per group) were used to analyze (A and B) effects of ibogalogs administration, (C and D) interaction between ketanserin (Keta) and ibogalogs, and (E and F) interaction between GR127935 (GR) and ibogalogs. Area under the curve (AUC) analyses performed by three-way ANOVA (drug, antagonist condition, and sex). Panels A-F: IBG *vs* vehicle, *p <0.05, **p <0.01, ***p <0.001; DM506 *vs* vehicle, ^p <0.05, ^^p <0.01, ^^^p <0.001; TBG *vs* vehicle, ^#^p <0.05, ^##^p <0.01, ^###^p <0.001.

Induction of the CSD model is reported to present in a sex dependent manner [7]. To assess potential sex-dependent effect of the ibogalogs on CSD, time-course von Frey behavioral data under CSD at different antagonist conditions were analyzed separately by sex (Figs. 3A-F; n = 5 per group). Within each sex, two-way ANOVA analysis revealed significant main effects of drug and time, as well as significant drug-by-time interactions (all p <0.01). AUC data were further analyzed using a three-way ANOVA including drug type, antagonist condition, and sex (Fig. 3G). This analysis showed significant main effects of antagonist condition [F_(2, 96)_ = 14.691; p <0.001; $\eta_{p}^{2}=0.23$] and drug [F_(3, 96)_ = 53.465; p <0.001; $\eta_{p}^{2}=0.63$] but not sex [F_(1,96)_ = 3.604; p =0.061; $\eta_{p}^{2}=0.04$]. No significant two-way or three-way interactions were observed (all p >0.05), further suggesting sex-independent effects of the ibogalogs.

### 3.2.a. Ibogalogs reduce MOH associated periorbital allodynia in female rats

**Figure 4.**
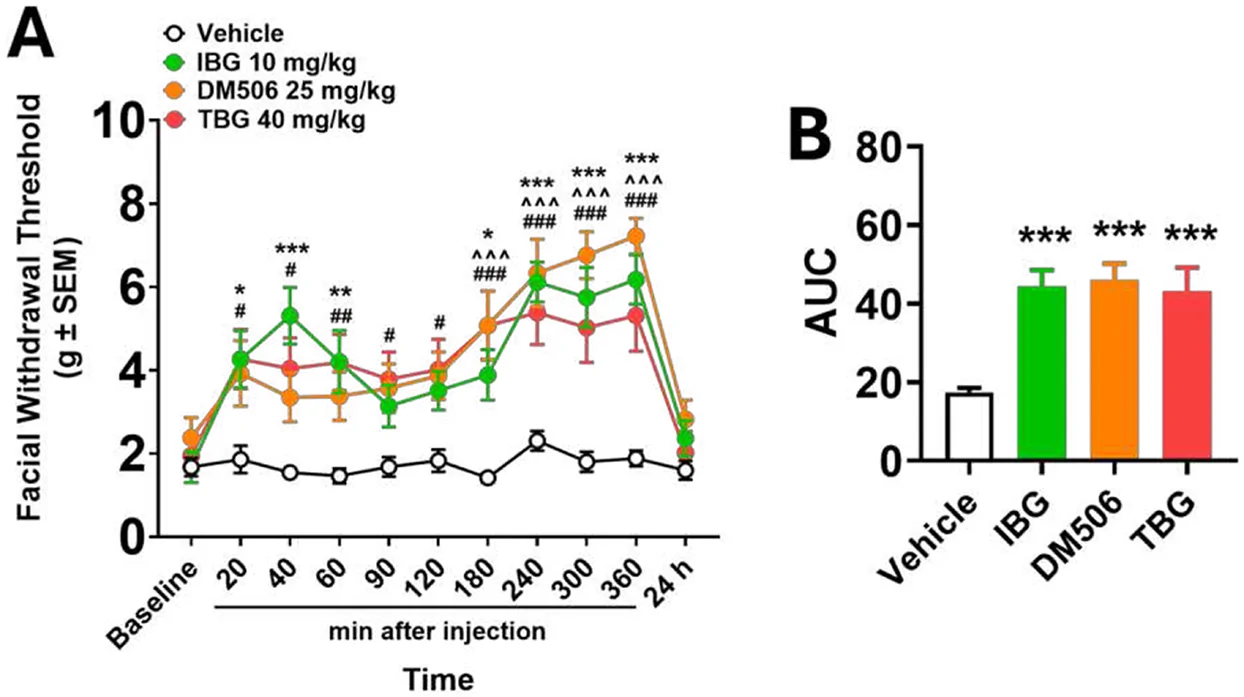

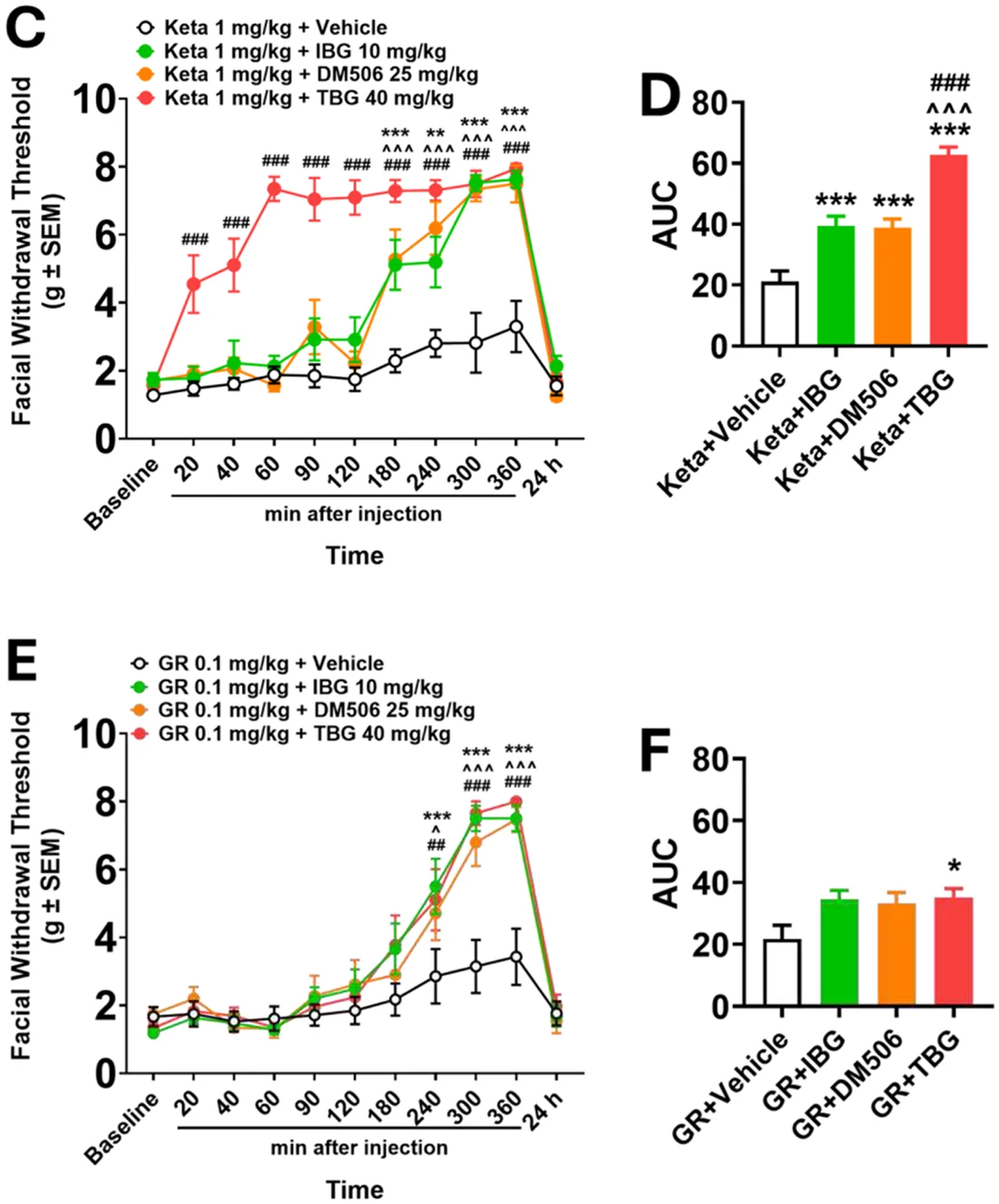
Periorbital von Frey testing in a sumatriptan-induced medication overuse headache (MOH) model in female rats. After 7 d of continuous sumatriptan infusion, rats were tested for baseline facial withdrawal thresholds. Subsequently, they received an intraperitoneal (i.p.) injection of 8.6 mg/kg IBG (base) (•), 21 mg/kg DM506 (base) (•), 26.6 mg/kg TBG (base) (•), or vehicle (○). Facial withdrawal thresholds were measured at 20, 40, 60, 90, 120, 180, 240, 300, and 360 min, and at 24 h post-injection. Data were analyzed by two-way ANOVA followed by Tukey’s post hoc test (mean ± SEM; n = 10 per group) for (A) effects of ibogalogs administration, (C) interaction between ketanserin (Keta) and ibogalogs, and (E) interaction between GR127935 (GR) and ibogalogs. Area under the curve (AUC) analyses performed by one-way ANOVA followed by Tukey’s post hoc test for (B) ibogalogs administration, (D) Keta–ibogalogs interaction, and (F) GR–ibogalogs interaction within 24 h. Panels A, C, and E: IBG *vs* vehicle, *p <0.05, **p <0.01, ***p <0.001; DM506 *vs* vehicle, ^p <0.05, ^^^p <0.001; TBG *vs* vehicle, ^#^p <0.05, ^##^p <0.01, ^###^p <0.001. Panels B, D, and F: ibogalogs *vs* vehicle, *p <0.05, ***p <0.001; TBG *vs* IBG, ^^^p <0.001; TBG *vs* DM506, ^###^p <0.001.

Initial results showed that rats developed periorbital mechanical allodynia after 7 d of sumatriptan infusion (Fig. 4A). To assess whether ibogalogs reduce sumatripan-induced facial allodynia, rats were administered TBG [26.6 mg/kg (base)], IBG [8.6 mg/kg (base)], DM506 [21 mg/kg (base)], or vehicle. Two-way ANOVA analysis showed significant main effects of drug [F_(3,36)_ = 9.519; p <0.001] and time [F_(10,360)_ = 27.033; p <0.001], as well as a significant drug-by-time interaction [F_(30,360)_ = 3.927; p <0.001]. Post-hoc Tukey’s tests compared to the vehicle group revealed that: (1) TBG markedly reduced periorbital allodynia throughout the 6-h observation period; (2) DM506 exerted a significant decrease within 90 min post-injection; and (3) IBG produced a bimodal effect pattern, with maximal effects at 40 min and between 4-6 h. The analgesic effects of all ibogalogs subsided at 24 h post-administration. AUC analysis further confirmed that ibogalogs significantly increased AUC values compared to the vehicle group with the same efficacy for all [F_(3,36)_ = 10.281; p <0.001] (Fig. 4B). These findings indicate that all three ibogalogs produce transient anti-allodynic effects in the MOH model with similar efficacy.

### 3.2.b. Ketanserin revealed divergent effects among different ibogalogs in reducing periorbital allodynia

To evaluate the involvement of 5-HT_2A/2C_Rs in the anti-allodynic effects of ibogalogs in MOH, rats were administered ketanserin (1 mg/kg) 30 min before ibogalog treatment (Fig. 4C). Two-way ANOVA analysis demonstrated significant main effects of drug [F_(3,36)_ = 30.291; p <0.001] and time [F_(10,360)_ = 73.606; p <0.001], as well as a significant drug-by-time interaction [F_(30,360)_ = 8.861; p <0.001]. Post-hoc Tukey’s analysis indicated that ketanserin abolished the effects of IBG during the first 2 h but enhanced the anti-allodynic effects of TBG. Three hours after ketanserin, all ibogalogs showed a similar pattern of periorbital allodynia reduction. AUC analysis revealed a significant difference among treatment groups [F_(3,36)_ = 31.336; p <0.001] (Fig. 4D). Post-hoc Tukey’s comparisons showed that ketanserin co-administration significantly increased the AUC for all ibogalogs relative to the ketanserin (alone) group (p <0.001), with TBG showing higher efficacy than IBG or DM506 (p <0.001; Fig. 4D). However, ketanserin did not induce any effect *per se* compared to the vehicle group [AUC unpaired T-test t_(18)_ =1.029; p >0.05]. These findings suggest that the ibogalog-induced attenuation of chronic headache-like allodynia is modulated *via* 5-HT_2_Rs.

### 3.2.c. GR127935 blocked the effects of ibogalogs in reducing MOH periorbital allodynia

To assess the role of 5-HT_1B/1D_R subtypes underlying the anti-allodynic effects of ibogalogs in MOH, rats were administered 0.1 mg/kg GR127935 30 min prior to ibogalog treatment to examine the receptor mechanism (Fig. 4E). Two-way ANOVA analysis identified significant main effects of drug [F_(3,36)_ = 3.016; p <0.05] and time [F_(10,360)_ = 105.424; p <0.001], as well as a significant drug-by-time interaction [F_(30,360)_ = 5.417; p <0.001]. Post-hoc Tukey’s tests showed that GR127935 abolished the anti-allodynic effects of ibogalogs during the first 3 h but not between 4-6 h. AUC analysis demonstrated a significant difference among the treatment groups [F_(3,36)_ = 3.430; p <0.05] (Fig. 4F). Post-hoc comparisons revealed that co-administration of GR127935 with TBG, but not with IBG or DM506, significantly increased AUC compared to the TBG group (p <0.041). However, GR127935 did not induce any effect *per se* compared to the vehicle group [AUC unpaired T-test: t_(18)_ = 0.946; p >0.05]. These results idicate that 5-HT_1B/1D_Rs are required for the early anti-allodynic effects of ibogalogs during MOH.

### 3.2.d. Effect of ibogalogs in MOH associated periorbital allodynia in wild type and 5-HT_1F_R KO mice

**Figure 5.**
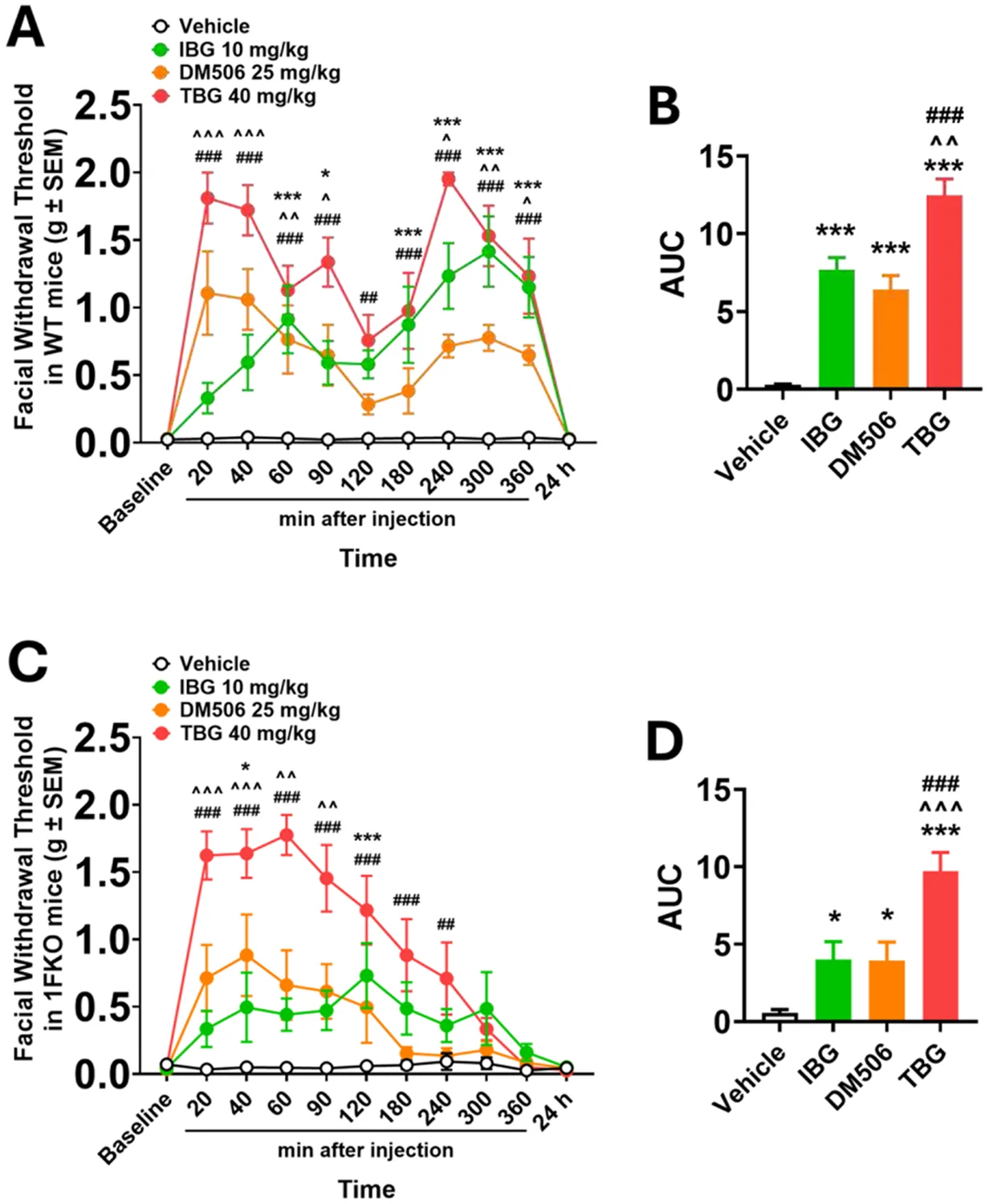
Periorbital von Frey testing in a sumatriptan-induced medication overuse headache (MOH) model in wild type and 5-HT_1F_R KO mice. After 7 d of continuous sumatriptan injection, mice were tested for baseline facial withdrawal thresholds. Subsequently, they received an intraperitoneal (i.p.) injection of 8.6 mg/kg IBG (base) (•), 21 mg/kg DM506 (base) (•), 26.6 mg/kg TBG (base) (•), or vehicle (○). Facial withdrawal thresholds were measured at 20, 40, 60, 90, 120, 180, 240, 300, and 360 min, and at 24 h post-injection. Data were analyzed by RM two-way ANOVA followed by Tukey’s post hoc test (mean ± SEM; n = 7) for effects of ibogalogs administration in (A) WT mice, and (C) 5-HT_1F_R KO mice. Area under the curve (AUC) analyses performed by RM one-way ANOVA followed by Tukey’s post-hoc test for (B and D) ibogalogs administration within 24 h. Panels A, and C: IBG *vs* vehicle, *p <0.05, ***p <0.001; DM506 *vs* vehicle, ^p <0.05, ^^^p <0.001; TBG *vs* vehicle, ^##^p <0.01, ^###^p <0.001. Panels B, and D: ibogalogs *vs* vehicle, *p <0.05, ***p <0.001; TBG *vs* IBG, ^^p <0.01, ^^^p <0.001; TBG *vs* DM506, ^###^p <0.001.

The results showed that both WT (Fig. 5A) and 5-HT_1F_R KO mice (n=7/genotype) (Fig. 5C) developed periorbital mechanical allodynia after 7 d of sumatriptan injections [Unpaired T-test t_(12)_ =1.133; p >0.05]. This indicates that 5-HT_1F_Rs are not playing a specific role in MOH induction.

To assess whether ibogalogs reduce established sumatriptan-induced facial allodynia, WT and KO mice were administered TBG [26.6 mg/kg (base)], IBG [8.6 mg/kg (base)], DM506 [21 mg/kg (base)], or vehicle with a within-subject design. RM two-way ANOVA analysis of the results in WT mice showed significant main effects of drug [F_(3,18)_ = 42.917; p <0.001] and time [F_(10,60)_ = 24.586; p <0.001], as well as a significant drug-by-time interaction [F_(30,180)_ = 4.804; p <0.001] (Fig. 5C). The post-hoc Tukey's test analysis of the results in WT mice revealed that TBG markedly reduced periorbital allodynia throughout the 6-h observation period compared with the vehicle group. The patterns of ibogalog-induced analgesia in mice (Fig. 4A) and rats (Fig. 5A) aligned with respect to duration of effect during the testing time-frame and bimodal peaks, indicating that all three ibogalogs produce anti-allodynic effects in both rodents. AUC analysis showed a significant difference among treatment groups [F_(3,18)_ = 42.783; p <0.001] (Fig. 5B). Post-hoc Tukey’s comparisons indicated that ibogalogs significantly increased AUC values compared to the vehicle group (p <0.001 for all), with TBG yielding a larger AUC than IBG (p <0.01) and DM506 (p <0.001), respectively.

Figure 5C illustrates the effect of ibogalogs using 5-HT_1F_R KO mice. RM two-way ANOVA analysis showed significant main effects of drug [F_(3,18)_ = 20.943; p <0.001] and time [F_(10,60)_ = 16.313; p <0.001], as well as a significant drug-by-time interaction [F_(30,180)_ = 6.922; p <0.001]. Post-hoc Tukey’s tests compared to the vehicle group revealed that (see Fig. 5C): (1) TBG markedly reduced periorbital allodynia throughout the 4-h observation period; (2) DM506 significantly decreased allodynia within the first 90 min post-injection; and (3) IBG significantly attenuated headache like behavior. The analgesic effects of all ibogalogs subsided at 5 h post-administration which was not observed in WT mice. AUC analysis revealed a significant difference among treatment groups [F_(3,18)_ = 21.343; p <0.001] (Fig. 5D). Post-hoc Tukey’s comparisons showed that TBG (p <0.001), IBG (p <0.05), and DM506 (p <0.05) increased the AUC relative to the vehicle group, where the effect of TBG was significantly larger than that for IBG or DM506 (p <0.001). Two-way ANOVA comparison of AUC values between WT and 5-HT_1F_R KO showed that loss of 5-HT_1F_R significantly attenuated the antiallodynic effect of ibogalogs independently with significant main effects of drug [F_(3,36)_ = 60.328; p <0.001] and genotype [F_(1,12)_ = 6.374; p = 0.027], but no significant interaction [F_(3,36)_ = 2.266; p = 0.098]. While AUC analysis reveals global reductions in efficacy across treatments without capturing temporal dynamics, further three-way ANOVA across time points was subsequently employed to capture the detailed interaction profiles and time-dependent effects of ibogalogs and gene modulation. Overall, these results showed that the anti-allodynic activity ibogalogs is attenuated in KO mice, supporting a role for the 5-HT_1F_R in MOH.

### 3.3 Temporal dynamics and interaction profiles of ibogalogs and serotonin receptors

**Figure 6.**
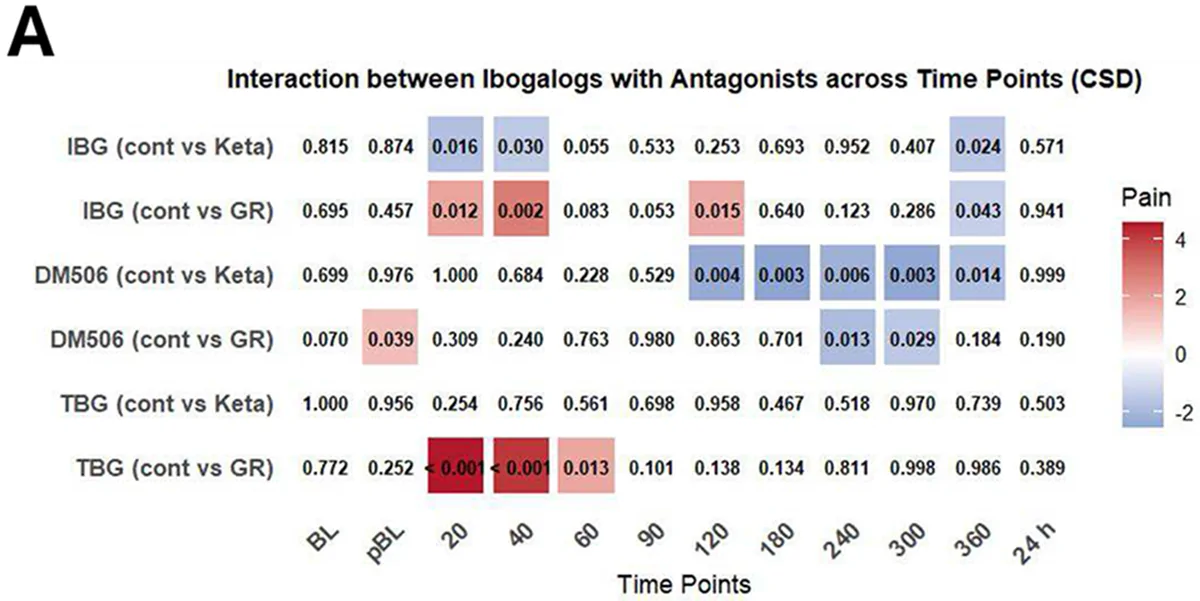

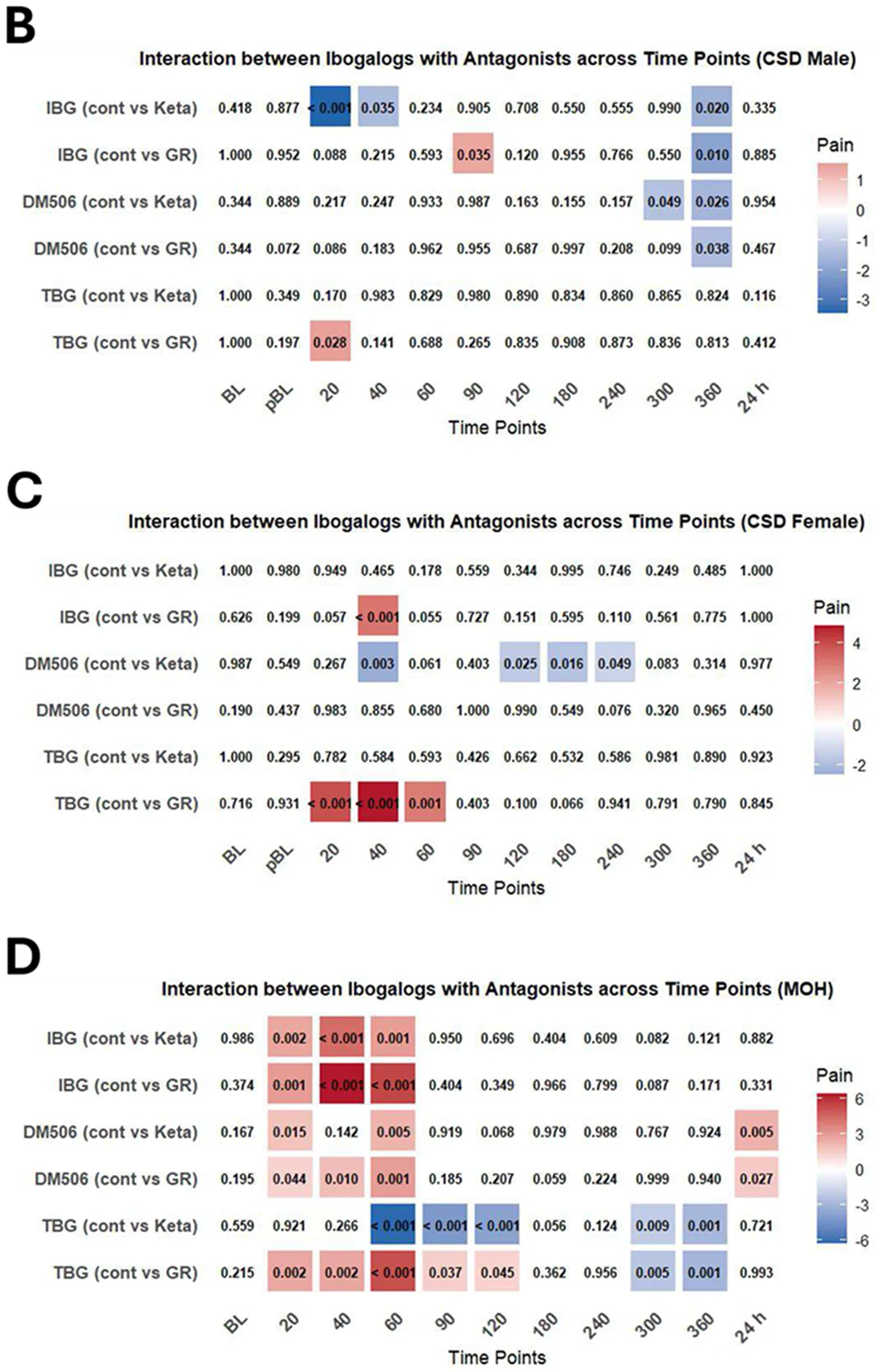

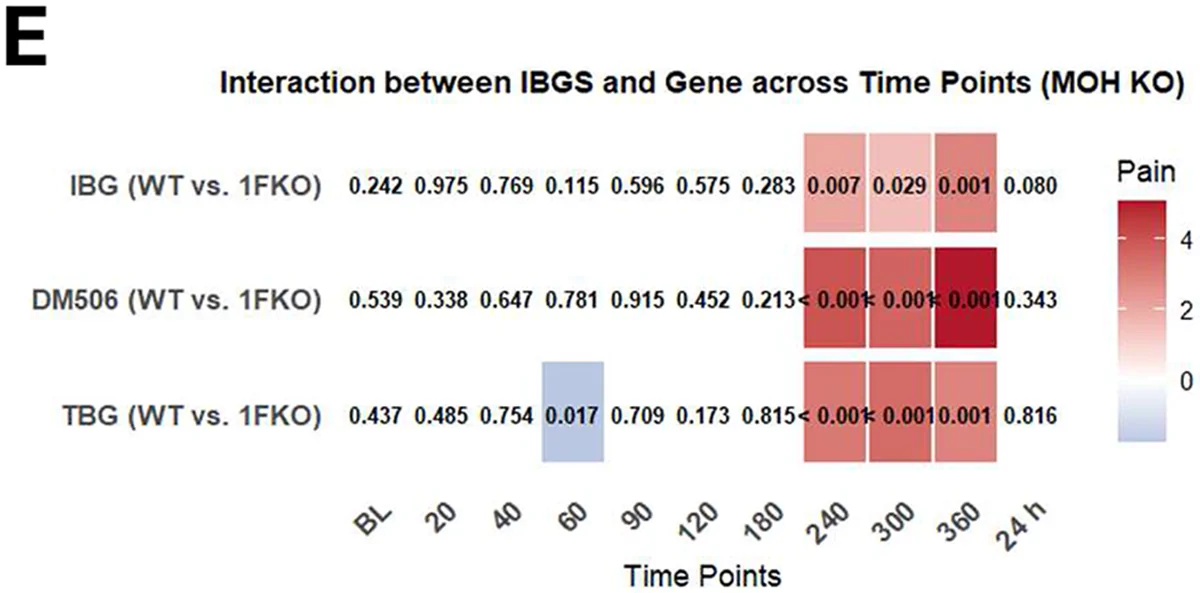
Heat maps illustrating the anti-headache activity of ibogalogs with 5-HT antagonists in rats and gene knockout mice across time points. Following three-way ANOVA, Tukey’s post-hoc test was performed to evaluate the interaction effects of ibogalogs and 5-HT antagonists in the CSD model for (A) both sexes and separated into (B) males, and (C) females, as well as (D) the MOH model in rats, and (E) the MOH model in wild-type (WT) and 5-HT_1F_R knockout (KO) mice. P-values are indicated, with the color spectrum representing pain levels ranging from high pain (red) to low pain (blue). Abbreviation: baseline (BL), post-cortical KCl injection baseline (pBL), ibogalogs alone control (cont), ketanserin (Keta), GR127935 (GR), WT mice (WT), and 5-HT_1F_R KO mice (1FKO).

To analyze the temporal dynamics of the interaction between ibogalogs and 5-HTRs, the three-way ANOVAs were used to evaluate the effects of ibogalogs-antagonists and ibogalogs-genotypes across different time points, which are illustrated in heat maps for rats in CSD (Fig. 6A-C) and in MOH (Fig. 6D), and for mice in MOH (Fig. 6E).

In CSD data, the three-way ANOVA revealed a significant drug-antagonist condition-time interaction [F_(66,1188)_ = 2.526; p <0.001] (Fig. 6A). Tukey's post-hoc analysis showed that ketanserin boosted the pain-relieved effects of IBG at 20, 40, and 360 min and of DM506 after 120 min, but not with TBG; however, GR127935 abolished the analgesia at the 40- and 60-min time points with IBG and TBG, respectively, but reduced pain responses with DM506 at the 240- and 300-min time points. Furthermore, this three-way interaction remained significant when the data were analyzed separately for male [F_(66,528)_ = 2.136; p <0.001] (Fig. 6B) and female rats [F_(66,528)_ = 1.347; p = 0.043] (Fig. 6C). In male rats, ketanserin enhanced the analgesic effects of IBG at 20, 40, and 360 min and of DM506 after 300 min, but again, not with TBG. On the other hand, GR127935 abolished the analgesia at 90 min with IBG and at 20 min with TBG, but reduced pain responses with IBG and DM506 at the 360-min time point. For female rats, ketanserin only boosted the pain-relieved effects with DM506 at 40 min and from 120 to 240 min. However, GR127935 induced more pain with IBG at 40 min and with TBG from 20 to 60 min.

For the MOH model, three-way ANOVA revealed a significant drug-antagonist condition-time interaction [F_(60,1080)_ = 2.902; p <0.001] (Fig. 6D). Tukey's post-hoc analysis showed that co-treatment with ketanserin increased the pain responses in rats treated with IBG or DM506 in the first 60 min, but decreased pain with TBG from 60 to 120 min and from 300 to 360 min. Similarly, GR127935 increased pain in rats treated with IBG and DM506 in the first 60 min, while the effect on TBG lasted until 120 min and prevented pain after 300 min. Three-way ANOVA analysis of the mice data showed a significant drug-genotype-time interaction [F_(30,360)_ = 2.467; p <0.001] (Fig. 6E). The 5-HT_1F_R KO mice showed higher pain responses than WT mice when treated with IBG, DM506, and TBG after 240 min, although a decrease in pain was observed at 60 min when treated with TBG. These findings highlight that the temporal dynamics of ibogalogs' analgesic effects are tightly regulated by distinct 5-HT receptor subtypes, exhibiting time-dependent profiles across different pain models.

### 3.4. Functional activity of ibogalogs at 5-HT_1B/1D/1F_R subtypes

**Figure 7.**
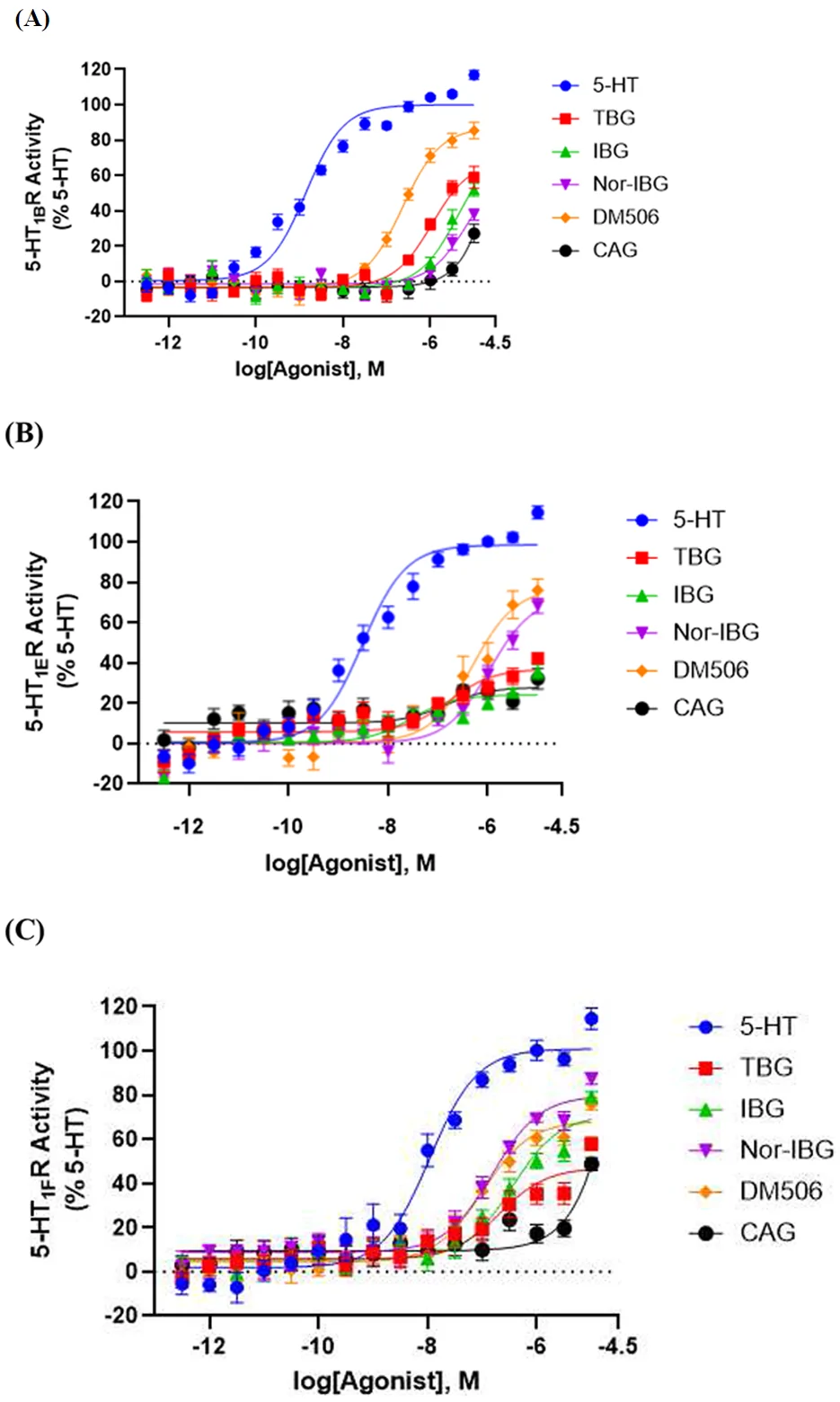
Functional activity of ibogalogs at the human 5-HT_1B/1E/1F_R subtypes. HEK293-T cells transfected with 5-HT_1B_R (A), 5-HT_1E_R (B), or 5-HT_1F_R (C) were incubated (30 min) with DM506 (◊), IBG (▲), nor-IBG (▼), CAG (•), or TBG (■) (0.1 nM-10 μM), and the activity compared to that for 5-HT (■). Concentration-response curves (mean ± SEM; n = 3-4) indicated that ibogalogs activate the 5-HT_1B_R (A), 5-HT_1E_R (B), and 5-HT_1F_R (C) with different potency (EC_50_) and efficacy (E_max_) values (listed in Table 1).

To determine the functional activity of ibogalogs at the human 5-HT_1B_R (Fig. 7A), 5-HT_1E_R (Fig. 7B), and 5-HT_1F_R subtypes (Fig. 7C), BRET assays were performed in HEK293-T cells expressing each receptor subtype. The concentration-response curves (mean ± SEM; n = 4) at the 5-HT_1B_R showed that the potency for 5-HT [EC_50_ (half-maximal stimulatory concentration) = 1.4 ± 0.3 nM] was higher than that for ibogalogs (Fig. 7A; Table 1). Nevertheless, some differences were observed among ibogalogs. DM506 (202 ± 29 nM) was more potent than TBG (703 ± 267 nM) in activating the 5-HT_1B_R, whereas IBG, nor-IBG, and CAG were much weaker (>10 μM) (Table 1). The observed ibogalog efficacies (E_max_) at 10 μM, compared to the maximal response for 5-HT (100 ± 11%), indicated that DM506 (91 ± 5%) is close to a full agonist, whereas TBG (69 ± 11%) acts as a partial agonist. Since the fitted curves for IBG, nor-IBG, and CAG did not reach saturation at concentrations up to 10 μM, their potencies and efficacies could not be determined with accuracy.

The concentration-response curves (mean ± SEM; n = 3) for ibogalogs at the 5-HT_1E_R showed overall lower potencies (EC_50_ = 1.1-2.0 μM) compared to that for the other 5-HT_1_R subtypes (Fig. 7B; Table 1). They acted as partial agonists, where DM506 (1,341 ± 923 nM; 85 ± 4%) and nor-IBG (1,352 ± 667 nM; 75 ± 10%) showed relatively higher potencies and E_max_ values (compared to the full agonist 5-HT (Fig. 7B; Table 1). Since the curve for CAG did not reach saturation at concentrations up to 10 μM, its potency and efficacy could not be determined with accuracy.

At the 5-HT_1F_R, most ibogalogs appear to be partial agonists with E_max_ values that range from ~43% to 73% compared to the full agonist 5-HT (Fig. 7C; Table 1). The concentration-response curves (mean ± SEM; n = 3) provided potencies that follow the sequence: DM506 (101 ± 14 nM) ~ nor-IBG (171 ± 76 nM) > IBG (469 ± 298 nM) ~ TBG (841 ± 703 nM) ≫ CAG (Fig. 7C; Table 1). Since the curve for CAG did not reach saturation at concentrations up to 10 μM, its potency and efficacy could not be determined with accuracy. Including previous data (Chagraoui et al., 2026), we were able to obtain the following receptor selectivity: 5-HT_1F_R ~ 5-HT_1D_R > 5-HT_1B_R ≫ 5-HT_1E_R ⋙ 5-HT_1A_R (no activity at >10 μM). This suggests structural divergences in the interaction with the orthosteric site of each 5-HT_1_R subtype, a topic that will be developed in future studies.

## 4. Discussion

In this study, we assessed whether ibogalogs such as IBG, DM506, and TBG reduce headache-like symptoms in rats after acute (KCl) and repeated treatments (sumatriptan) using the respective CSD and MOH models, and potential sex-dependence using the CSD model. We also assessed the role of different 5-HT_1_R and 5-HT_2_R subtypes in their effects using selective antagonists, as well as 5-HT_1F_R KO mice. In addition, we determined the functional activity of a variety of ibogalogs at the respective 5-HT_1D/1E/1F_R subtype.

The CSD results showed that TBG and IBG, but not DM506, decrease KCl-induced facial allodynia, an effect that lasted for 6 h. These results could be easily explained considering that TBG and IBG, but not DM506, functionally behave as competitive antagonists of the 5-HT_2B_R [18, 19]. It is very well documented that compounds that inhibit the 5-HT_2_R, 5-HT_3_R, or 5-HT_7_R subtype may prevent or reduce the occurrence of migraine attacks in rodents and human beings ([37, 38]; reviewed in [3]). Functional results showed that ibogalogs practically do not affect the 5-HT_3_R and act as weak inverse agonists at the 5-HT_7_R [14, 16], ruling out the involvement of these receptor subtypes in the anti-allodynic activity of ibogalogs. The anti-allodynic effects of ibogalogs were sex independent in the CSD model despite the model may discern sex dependent phenotypes [7]. Additional studies are warranted to evaluate sex-dependent regulation of 5-HTR subtypes in this model.

Based on the CSD results, it is possible that TBG/IBG-induced 5-HT_2B_R inhibition may decrease acute allodynia. In meningeal blood vessels, activation of 5-HT_2B_Rs expressed in endothelial cells increases nitric oxide, which can induce migraine attacks [39]. However, when ketanserin was co-injected with each ibogalog, enhancement, but not inhibition, of the anti-allodynic activity was observed. A potential explanation is that ketanserin blocks the stimulatory activity of ibogalogs at 5-HT_2A/2C_Rs, without modifying the inhibitory activity of TBG/IBG at the 5-HT_2B_R, finally producing more complete inhibition of these receptors and consequently more efficient anti-allodynic effect. Given that ketanserin binds to the 5-HT_2A_R (K_i_ = 1.13 nM) with higher affinity than that at the 5-HT_2C_R (K_i_ = 88 nM) and 5-HT_2B_R (K_i_ = 233 nM) supports such idea. Indeed, ketanserin induced anti-nociceptive activity (ED_50_ ~0.6-1.5 mg/kg, depending on the used test) likely *via* supraspinal 5-HT_2_R-evoked inhibition of descending nociceptive neurotransmission [2]. However, it does not explain that the least active ibogalog, DM506, improved its overall efficacy between 2-6 h post-injection. Our CSD and MOH results showed that ketanserin does not induce any effect *per se*, supporting previous studies [40].

A hypothetical scenario for the anti-acute headache activity of ibogalogs can be based on previous studies with triptans showing that presynaptic 5-HT_1D_R activation induces cranial vasoconstriction and decreases the release of inflammatory neuropeptides such as calcitonin receptor peptide (CGRP) from the trigeminal sensory nerve ending, whereas 5-HT_1D/1E_R activation may also inhibit trigeminal nuclei excitability ([24]; reviewed in [41]). Unfortunately, our behavioral study cannot discern ibogalog activity between 5-HT_1B/1D_Rs expressed in vascular smooth muscles and trigeminal neurons. However, we were able to determine the functional activity of several ibogalogs at these receptor subtypes *in vitro*. These and previous results [20] showed that the potency sequence at the 5-HT_1B/1D/1E_Rs (DM506 higher than TBG/IBG) does not support the efficacy observed in our behavioral tests, where IBG/TBG were more efficient than DM506. In this regard, we assessed the role of 5-HT_1B/1D_Rs using the selective antagonist GR127935. Our results showed that this antagonist mitigates the anti-allodynic effects of ibogalogs observed between 1-3 h but not after 3 h. These results aligned with a previous study showing that GR127935 reduces zolmitriptan-induced 5-HT_1B/1D_R stimulation with the subsequent inhibition of trigeminal ganglion neurons [42]. According to previous results, GR127935 was unable to modulate presynaptic receptors [43], ruling out this potential negative feedback mechanism.

The MOH results showed that repeated sumatriptan treatment induces chronic headache-like periorbital allodynia, as observed in experimental animals and human beings [5, 44-47]. Although ibogalogs showed a bimodal profile across the experimental duration, all tested compounds significantly reduced chronic headache with similar efficacy, which lasted for up to 6 h. This is an important difference with the CSD results, where DM506 failed to decrease acute allodynia. The observed differences may arise because prolonged use of sumatriptan overstimulates 5-HT_1B/1D_Rs on primary afferent neurons, producing neuronal sensitization, while 5-HT_1B/1D_R inhibition completely prevents such process. Sumatriptan-evoked overstimulation induces protein kinase Cε-dependent type I hyperalgesic priming, finally enhancing CGRP, pituitary adenylate cyclase-activating polypeptide (PACAP), and nitric oxide synthase (NOS) signaling, among other effects [44-47].

There is ample information indicating functional crosstalk between 5-HT_1_R and 5-HT_2_R subtypes in neuronal and non-neuronal cells [48-50]. For instance, the excitatory activity mediated by 5-HT_1A_R activation is reduced in the presence of 5-HT_2B_Rs, whereas 5-HT_2A_R activation decreased 5-HT_1A_R-agonist activity, and 5-HT_1B_R activation accelerated 5-HT_2B_R internalization. Considering the existence of a co-regulatory mechanism between 5-HT_2A_R and 5-HT_1B/1D_R, a possible ibogalog-driven mechanism might be occurring, where 5-HT_2A_R activation decreases sumatriptan-mediated 5-HT_1B/1D_R overstimulation. Our results in the CSD model showed that ketanserin enhanced the anti-allodynic effects of ibogalogs, while in the MOH model only TBG actions were enhanced. In contrast, GR127935 induces a clearer inhibitory effect in both models. GR127935-induced 5-HT_1B/1D_R inhibition may counteract the allodynic activity mediated by chronic sumatriptan stimulation, improving further the antiallodynic activity of ibogalogs *via* 5-HT_2A_R activation. The enhancing effect of ketanserin, on the other hand, might be based on its inhibitory action on upregulated 5-HT_2A_Rs observed during chronic headache (Zheng et al., 2020). These mechanisms will be tested in future studies.

Pharmacokinetics, metabolic, and/or pharmacodynamic features might be implicated in the higher efficacy of TBG/IBG *vs* DM506 in the CSD experiments and bimodal time-course in MOH. Based on similar lipophilicity (LogP = 2.5-2.6) and brain penetration (LogBBB = 0.62-0.75) features for TBG, IBG, and DM506 (Arias et al., 2025b), we can discard pharmacokinetics differences. We also know that TBG (50 mg/kg; i.p.) rapidly reaches the brain (~15 min) and remains for ~3 h [14]. However, we do not know the metabolism for IBG or DM506. Based on their physicochemical parameters, we expect different receptor interactions as observed in 5-HT_2_R subtypes [19, 51]. As an attempt to address possible pharmacodynamics differences among ibogalogs, the functional activity of TBG, DM506, IBG, nor-IBG, and CAG was determined at the 5-HT_1B_R, 5-HT_1E_R, and 5-HT_1F_R subtypes, complementing previous studies at the 5-HT^1A^R and 5-HT_1D_R subtypes [20]. The functional assays showed that ibogalogs are in general partial agonists at different 5-HT_1_R subtypes. Regarding ibogalog potency at each receptor subtype, the following sequence for the most efficient compound, DM506, was observed: 5-HT_1F_R (101 ± 14 nM) ~ 5-HT_1D_R (110 ± 20 nM) > 5-HT_1B_R (202 ± 29 nM) ≫ 5-HT_1E_R (1,341 ± 923 nM) ⋙ 5-HT_1A_R (>10 μM). Based on our functional results showing a comparatively lower activity for ibogalogs at the 5-HT_1E_R, a nil activity of IBG at the 5-HT_1B_R, but a potent agonistic activity at the 5-HT_1D_R and 5-HT_1F_R, we suggest a potential role for the latter two receptor subtypes in the anti-headache activity of ibogalogs. Thus, to assess the potential role of the 5-HT_1F_R, we compared the anti-allodynic activity of ibogalogs between WT and 5-HT_1F_R KO mice. The results showed that the anti-allodynic activity ibogalogs in the MOH model is attenuated in KO mice at later timepoints. This indicates a time-dependent role for the 5-HT_1F_R in the activity of ibogalogs.

The role of 5-HT_1F_R in migraine therapy has gained significant attention due to the clinical application of lasmiditan. Unlike triptans, which act as 5-HT_1B/1D_R agonists and induce vasoconstriction, the 5-HT_1F_R agonist lasmiditan achieves therapeutic efficacy without associated cardiovascular side effects [52]. Like 5-HT_1B/1D_Rs, the 5-HT_1F_R has been shown to be expressed in the brain (reviewed in [53]), cerebrovascular tissues [54], and peripheral arteries [55]. The current data support the idea that the 5-HT_1F_R plays a role in headache subtypes (e.g., medication overuse headache). However, the specific mechanisms underlying the anti-migraine effects of 5-HT_1F_R activation remain poorly understood.

In conclusion, ibogalogs can alleviate acute headache-like behaviors in a sex-independent manner as well as chronic migraine induced by sumatriptan. The antagonist findings suggest that ibogalogs decrease acute headache-like symptoms by a co-modulatory mechanism involving both 5-HT_2_R and 5-HT_1_R subtypes, whereas chronic headache is reduced by co-modulatory mechanisms that also include the 5-HT_1F_R. This work provides strong evidence to support future studies to investigate the dynamics of 5-HTR expression and signaling in acute and chronic headache models and use of ibogalogs in the treatment of headache.

## Figures and Tables

**Table 1.** Functional activity of ibogalogs at different 5-HT_1_R subtypes.

Receptor Subtype	Compound	EC_50_ (nM)	E_max_ (%)
5-HT_1B_R	5-HT	1.4 ± 0.3	100 ± 11
	DM506	202 ± 29	91 ± 5
	TBG	737 ± 267	69 ± 11
	IBG	>10 μM	~50
	Nor-IBG	>10 μM	~40
	CAG	>10 μM	~40
5-HT_1D_R^a^	5-HT	1.7 ± 0.3	100 ± 18
	DM506	110 ± 20	89 ± 3
	TBG	209 ± 58	61 ± 7
	IBG	661 ± 182	59 ± 12
	Nor-IBG	447 ± 134	75 ± 7
	CAG	407 ± 164	40 ± 6
5-HT_1E_R	5-HT	5.9 ± 4.3	100 ± 0
	DM506	1,341 ± 923	85 ± 4
	TBG	1,119 ± 739	35 ± 5
	IBG	2,043 ± 1,698	38 ± 10
	Nor-IBG	1,352 ± 667	75 ± 9
	CAG	>10 uM	~20
5-HT_1F_R	5-HT	12 ± 5	100 ± 18
	DM506	101 ± 14	66 ± 7
	TBG	841 ± 703	43 ± 11
	IBG	469 ± 298	67 ± 10
	Nor-IBG	171 ± 76	73 ± 9
	CAG	>10 μM	~43

Values are expressed as the mean ± SEM.

The EC_50_ and E_max_ values were obtained from Figure 7.

The E_max_ percentages were calculated considering 100% activation by 5-HT.

a Values (taken from Chagraoui et al., 2026) included for comparative purposes

## Abbreviations:

5-HT: serotonin
5-HT_x_R: serotonin receptor subtypes (x = 1A, 1B, 1D, 1E, 1F, 2A, 2B, 2C)
GABA: γ-aminobutyric acid
MOH: medication overuse headache
CSD: cortical spreading depression
DM506 (ibogaminalog): 3-methyl-1,2,3,4,5,6-hexahydroazepino[4,5-*b*]indole
TBG (tabernanthalog): 8-methoxy-3-methyl-1,2,3,4,5,6-hexahydroazepino[4,5-*b*]indole
IBG (ibogainalog): 9-methoxy-3-methyl-1,2,3,4,5,6-hexahydroazepino[4,5-*b*]indole
nor-IBG: 3-methyl-1,2,3,4,5,6-hexahydroazepino[4,5-*b*]indol-9-ol
CAG (catharanthalog): methyl 3-methyl-2,4,5,6-tetrahydro-1*H*-azepino[4,5-*b*]indole-5-carboxylate
BRET: bioluminescence resonance energy transfer
RT: room temperature
